# Identification and characterization of influenza A viruses in selected domestic animals in Kenya, 2010-2012

**DOI:** 10.1371/journal.pone.0192721

**Published:** 2018-02-09

**Authors:** Peninah Munyua, Clayton Onyango, Lydia Mwasi, Lilian W. Waiboci, Geoffrey Arunga, Barry Fields, Joshua A. Mott, Carol J. Cardona, Philip Kitala, Philip N. Nyaga, M. Kariuki Njenga

**Affiliations:** 1 Division of Global Health protection, United States Centers for Disease Control and Prevention-Kenya, Nairobi, Kenya; 2 Faculty of Veterinary Medicine, University of Nairobi, Nairobi, Kenya; 3 Center for Global Health Research, Kenya Medical Research Institute, Nairobi, Kenya; 4 National Center for Immunization and Respiratory Diseases, United States Centers for Disease Control and Prevention, Atlanta, Georgia, United States of America; 5 Department of Veterinary and Biomedical Sciences, University of Minnesota, St. Paul, Minnesota, United States of America; South China Agricultural University, CHINA

## Abstract

**Background:**

Influenza A virus subtypes in non-human hosts have not been characterized in Kenya. We carried out influenza surveillance in selected domestic animals and compared the virus isolates with isolates obtained in humans during the same period.

**Methods:**

We collected nasal swabs from pigs, dogs and cats; oropharyngeal and cloacal swabs from poultry; and blood samples from all animals between 2010 and 2012. A standardized questionnaire was administered to farmers and traders. Swabs were tested for influenza A by rtRT-PCR, virus isolation and subtyping was done on all positive swabs. All sera were screened for influenza A antibodies by ELISA, and positives were evaluated by hemagglutination inhibition (HI). Full genome sequencing was done on four selected pig virus isolates.

**Results:**

Among 3,798 sera tested by ELISA, influenza A seroprevalence was highest in pigs (15.9%; 172/1084), 1.2% (3/258) in ducks, 1.4% (1/72) in cats 0.6% (3/467) in dogs, 0.1% (2/1894) in chicken and 0% in geese and turkeys. HI testing of ELISA-positive pig sera showed that 71.5% had positive titers to A/California/04/2009(H1N1). Among 6,289 swabs tested by rRT-PCR, influenza A prevalence was highest in ducks [1.2%; 5/423] and 0% in cats and turkeys. Eight virus isolates were obtained from pig nasal swabs collected in 2011 and were determined to be A(H1N1)pdm09 on subtyping. On phylogenetic analysis, four hemagglutinin segments from pig isolates clustered together and were closely associated with human influenza viruses that circulated in Kenya in 2011.

**Conclusion:**

Influenza A(H1N1)pdm09 isolated in pigs was genetically similar to contemporary human pandemic influenza virus isolates. This suggest that the virus was likely transmitted from humans to pigs, became established and circulated in Kenyan pig populations during the study period. Minimal influenza A prevalence was observed in the other animals studied.

## Introduction

Influenza A viruses circulate in many animal species including domestic and wild birds, pigs and humans [1]. Interspecies transmission of influenza A viruses is common among different animal species via direct or indirect contact [1]. Whereas water birds appear to be the reservoir of all influenza A viruses, pigs play a key role in the evolution and emergence of novel influenza strains with pandemic potential [2, 3]. Importantly, transmission and maintenance of human origin influenza virus strains in pig populations raises the possibility of genetic reassortment with swine influenza viruses that could result in emergence of novel influenza virus strains with pandemic potential [2, 3]. In addition, avian species harbor novel influenza viruses that are subsequently transmitted to mammalian hosts including humans [4]. Thus, monitoring influenza viruses circulating in pig, avian and other animal populations is important for early detection of emerging strains with pandemic potential.

In Kenya, influenza A has been reported in poultry [5], but the subtypes circulating in poultry and other domestic animals have not been characterized. This study aimed to identify and characterize influenza viruses in chickens, ducks, geese, turkeys, cats, dogs and pigs in households in Nairobi and in Siaya County, as well as pigs presented for slaughter near Nairobi. Additionally, genetic comparison of influenza viruses isolated from pigs with viruses obtained from humans in Kenya was done to infer transmission patterns.

## Materials and methods

### Study site and sample collection

Repeated cross-sectional sampling was carried out among pigs, poultry, dogs and cats at the household level and among pigs presented for slaughter at a slaughterhouse between 2010 and 2012.

### Household based sampling

**Fig 1:**
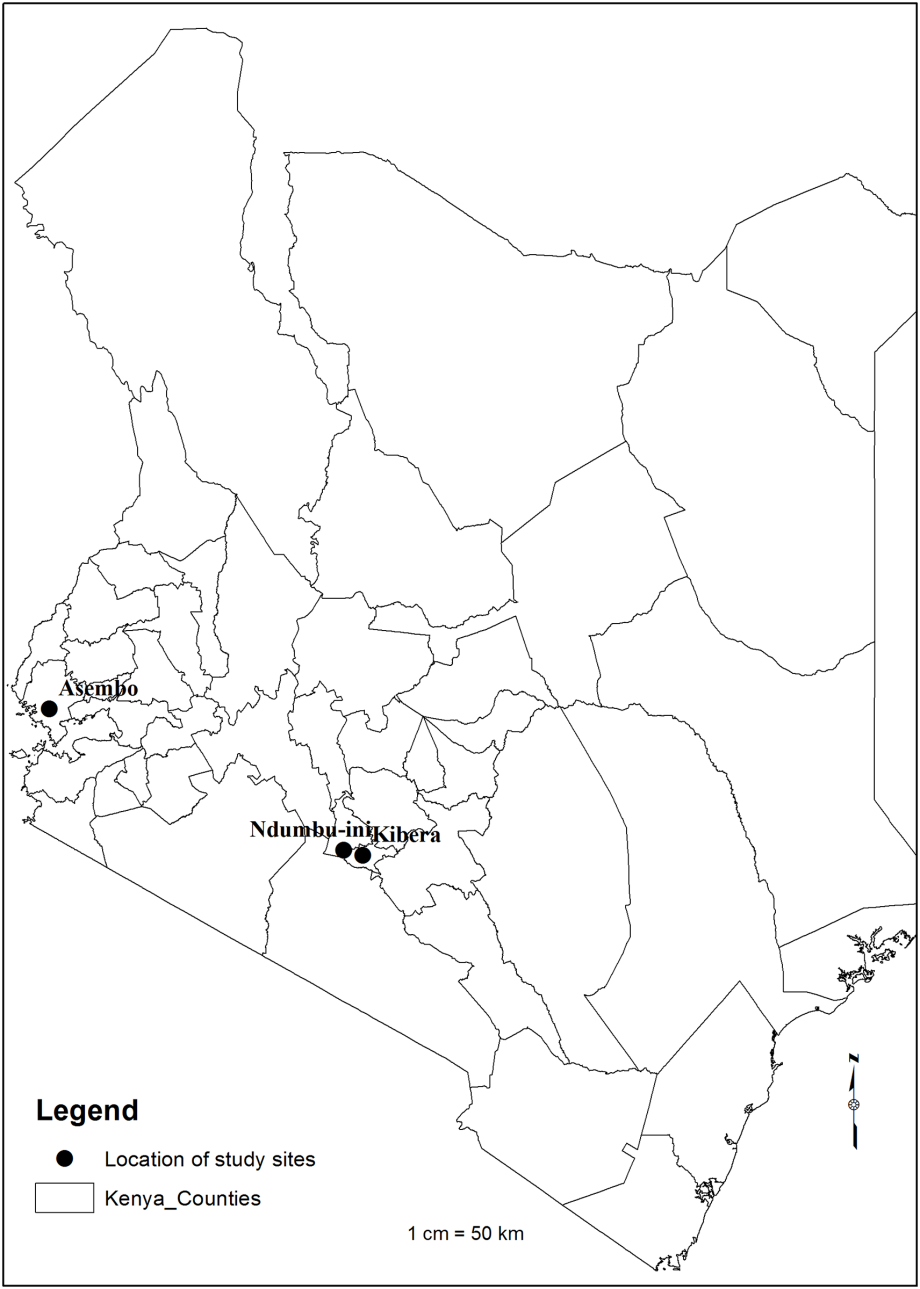
Map of Kenya counties showing the location of the study sites, Kibera in Nairobi, Ndumbui-ini slaughterhouse in Kiambu County and Asembo in Siaya County.

Sampling was conducted at two study sites where the Centers for Disease Control and Prevention—Kenya (CDC), in partnership with the Kenya Medical Research Institute (KEMRI), has conducted population-based infectious disease surveillance since 2007: Asembo, a rural location in western Kenya along Lake Victoria; and Gatwikira and Soweto villages within Kibera, an urban informal settlement in Nairobi [6, 7] (Fig 1). Sampling was carried out once in May 2010 in 103 randomly selected households in Kibera; in August 2011, 110 households were randomly selected from a list of households enrolled in the surveillance in Kibera and Asembo and sampled every three months through August of 2012. A standardized questionnaire was administered to an adult member of each household to record the species and number of animals present and the number of specimens collected. In each of the selected households, a convenience sample of a maximum of three for each of the animals (chickens, ducks, geese, turkeys, dogs and cats) and a maximum of 15 pigs were selected and specimens collected. There was no mechanism to record which specific animals had been sampled, and so on repeat visits some specimens represent re-sampling of animals that had been sampled on a prior visit.

### Slaughter house sampling

Specimens were collected from pigs presented for slaughter at a facility located on the outskirts of Nairobi in May 2010 and every three months between August 2011 and December of 2012. A maximum of four pigs delivered by a trader or farmer were sampled by convenience selection. Blood samples and nasal swabs were collected from all pigs sampled while bronchial swabs were collected from a subset of pigs. A standardized questionnaire was administered to the trader or farmer of the selected pigs to collect information on the source of the pigs, the number of pigs brought for slaughter and number of pigs present at the farms of origin.

### Sample collection and storage

Nasal and cloacal swabs were collected using plastic shafted polyester tipped swabs. Swabs were put into 2ml cryovials (Greiner Bio-One^®^, Germany) containing 1ml of viral transport media containing bovine serum albumin and veal infusion broth supplemented with amphotericin B and gentamycin (www.who.int/csr/resources/publications/surveillance/Annex8.pdf). and transported on ice to the laboratory. Blood samples were transported to the laboratory for sera harvesting. All specimens submitted to the laboratory in Nairobi were immediately frozen at -80°C and transported to Kisumu. All samples were tested at the KEMRI/CDC BSL-3 laboratory in Kisumu, Kenya.

### Laboratory testing

#### ELISA testing

Sera were tested for antibodies against influenza A viruses using the IDEXX^®^ ELISA (FlockChek AI MultiS-Screen Ab Test Kit^®^, Westbrook, Maine). The manufacturer recommended sample value to the negative control value (S/N) ratio cut-off of ≤0.5 was applied for poultry, cat and dog sera. An adjusted cut-off of the S/N of <0.673 was applied for pig sera in order to increase test sensitivity and specificity, as previously described [8].

#### Hemagglutination inhibition testing for pigs, dogs and cats sera

All ELISA-positive pig sera were also tested by hemagglutination inhibition (HI), as described [9], for antibodies to three influenza A virus strains: A/California/04/2009(H1N1), A/Swine/Texas/4199-2/98 triple-reassortant (H3N2) and A/Swine/Iowa/15/30 (H1N1); all obtained from St. Jude Children’s Research Hospital, TN, USA. A/California/04/2009 (H1N1) is an early 2009 pandemic virus; A/Swine/Texas/4199-2/98(H3N2) and A/Swine/Iowa/15/30 (H1N1) were first isolated in swine in the US in 1998 and 1930 respectively [10, 11]. Since the identity of swine influenza viruses circulating in Kenyan pigs is unknown these parent virus strains of the representative antigens were selected. Positive pig sera obtained from University of Minnesota St. Paul, Minnesota, USA was used in all experiments. Guinea pig red blood cells were used for the HI. Since appropriate reference antigens for poultry, dogs and cats were not available, sera from these animals were only tested against the A/California/04/2009(H1N1). Titers greater than or equal to 1:80 were considered positive.

#### Real-time reverse transcriptase polymerase chain reaction

All swabs were tested for influenza A virus RNA by real time reverse transcription-polymerase chain reaction (rRT-PCR) using primers and probes that target the matrix gene of all influenza A viruses [12]. Cut-off for positivity was read at cycle threshold (C_T_) values ≤39.9. Appropriate negative and positive control specimens were run alongside each reaction. Viral subtyping was performed on all isolates from pigs. RNA from the isolates was subtyped by rRT-PCR using primers and probes that were designed to target the hemagglutinin and Neuraminidase genes (H1, H3, N1 and N2) of North American swine influenza viruses [13].

#### Virus isolation

Virus isolation was attempted on rRT-PCR positive specimens and a proportion of rRT-PCR negative specimens by inoculation in embryonated chicken eggs for the poultry samples or in Madin-Darby Canine Kidney (MDCK) cells for mammalian samples. All virus isolation procedures were performed as described elsewhere [14].

#### Phylogenetic analysis of the HA genes

Full genome sequencing of the virus isolates was performed by the Virology, Surveillance and Diagnosis Branch, Influenza Division, CDC, Atlanta (S1 Table). Homologs of HA genes of the Kenya swine isolates were obtained through BLAST searches in the Influenza Research Database for samples collected between 2009 and 2011. A list of isolates selected from the BLAST search and included in the phylogenetic analysis is provided (S2 Table).

HA nucleotide contigs were assembled using the SeqMan Pro of Lasergene 11 software (DNASTAR, Inc, Madison, WI, US) and protein translations were done in Seqbuilder of Lasergene 11 software (DNASTAR, Inc, Madison, WI, US). Multiple sequence alignments were performed in BioEdit [15]. Phylogenetic analyses were performed with MrBayes 3.1 [16]. The generalized time-reversible model parameters and priors were incorporated into the nexus file for execution in MrBayes. The data were executed in MrBayes by running 1 million Monte Carlo Markov chains with a sampling frequency of 1000. The trees were rooted with A/California/04/09, a virus isolated from a human at the start of the 2009 influenza pandemic. The phylogenetic tree was edited in Inkscape ver 1.1 [17]

### Ethical approval

The protocol and sample collection procedures were reviewed and approved by the Animal Care and Use Committee (ACUC) and the Ethical Review Committee at KEMRI (SSC #1191), the Institutional ACUC (#1562) and Institutional Review Board of the U.S. CDC (#5410) and the University of Minnesota IACUC (#1002A77152). Informed consent was obtained from all participants (household heads and traders) whose animals were sampled. The collection of blood and swab samples from animals was always supervised by a veterinarian. Animals were handled in manner aiming to minimize stress and suffering, and were only restrained for the shortest period of time necessary.

### Statistical analysis

Univariate analyses were carried out to describe demographic characteristics of the animal populations sampled. The study outcome was influenza A virus prevalence and sero-prevalence for each of the sampling periods by animal type with 95% confidence intervals reported for these estimates. Chi square tests were used to compare the observed sero-prevalence across the sampling period and p-values were reported at <0.05 significance level. Stata statistical software (StataCorp, College Station, TX) was used for all statistical analyses.

## Results

### Influenza A seroprevalence by ELISA at the household level

In 2010, 103 households were enrolled in Kibera. In 2011, 110 households were enrolled in Kibera and 111 households were enrolled in Asembo. In Asembo, almost all (96.4%) of the households owned chickens (Table 1). None of the households in Asembo owned pigs. In Kibera, three quarters of the households (75.5%) owned chickens.

**Table 1:** Proportion of enrolled households (HH) owning different animal types by study site, Kenya, May 2010-August 2012.

Animal type	Asembo		Kibera	
	N = 111		N = 110	
	Number (%) HHs owning	Mean number* (sd)	Number (%) HHs owning	Mean number* (sd)
Cats	54 (48.6)	1.5 (1.5)	32 (29.1)	1.3 (0.7)
Cattle	77 (69.4)	7.6 (10.0)	2 (1.8)	4.0 (1.4)
Chickens	107 (96.4)	11.5 (11.3)	83 (75.5)	6.2 (7.3)
Dogs	64 (57.7)	1.8 (1.1)	27 (24.5)	2.9 (2.5)
Ducks	9 (8.1)	22.3 (28.3)	22 (20.0)	15.0 (4.3)
Goats	68 (61.3)	7.3 (12.3)	2 (1.8)	4.5 (2.1)
Pigs	0 (0)	-	4 (3.6)	27.8 (1.4)
Turkeys	6 (5.4)	24.8 (28.4)	4 (3.6)	17.2 (28.5)

*Mean number of specified animal, among households owning that animal type.

“sd” denotes standard deviation.

A total of 2,841 sera including 1,516 (53.4%) from Kibera and 1,325 (46.6%) from Asembo were tested for influenza A virus antibodies (Table 2). In Kibera, the influenza A seroprevalence was highest among pigs at 10.2% (n = 13), followed by cats at 2.1% (n = 1), dogs at 1.3% (n = 3) and chicken 0.2% (n = 2). All sera collected from geese (n = 2) and turkeys (n = 18) were negative for influenza virus antibodies. All sera collected from animals in Asembo were negative influenza A virus antibodies.

**Table 2:** Seroprevalence of serum influenza A antibodies by ELISA, by study site and animal type, Kenya, May 2010-August 2012.

Animal type	Study site				Total		
	Kibera		Asembo‡				
	Total samples tested	No. positive (%)	Total samples tested	No. positive (%)	samples tested	No. positive (%)	Binomial Exact 95% CI
Cat	47	1 (2.1)	25	0 (0)	72	1 (1.4)	0.03, 7.5
Chicken	855	2 (0.2)	1039	0 (0)	1894	2 (0.1)	0.01, 0.3
Dog	237	3 (1.3)	230	0 (0)	467	3 (0.6)	0.1, 1.9
Duck	230	3 (0.4)	28	0 (0)	258	3 (1.2)	0.2, 3.3
Goose	2	0 (0)	0	-	2	0 (0)	88.8*
Pig	127	13 (10.2)	0	-	127	13 (10.2)	5.5, 16.9
Turkey	18	0 (0)	3	0 (0)	21	0 (0)	18.8*
**Total**	**1516**	**25 (1.6)**	**1325**	**0 (0)**	**2841**	**25 (0.9)**	**0.6, 1.3**

ELISA denotes enzyme-linked immunosorbent assay

^‡^ In Asembo samples were not collected in May 2010

*one-sided, 97.5% confidence interval reported

In pigs sampled in Kibera households, the seroprevalence was 8.9% (5/56) in May 2010, 28.0% (7/25) in August 2011 and 2.8% (1/35) in December 2011. None of the sera collected from pigs in April and August of 2012 was positive.

### Influenza A ELISA seroprevalence in slaughterhouse pigs

**Fig 2:**
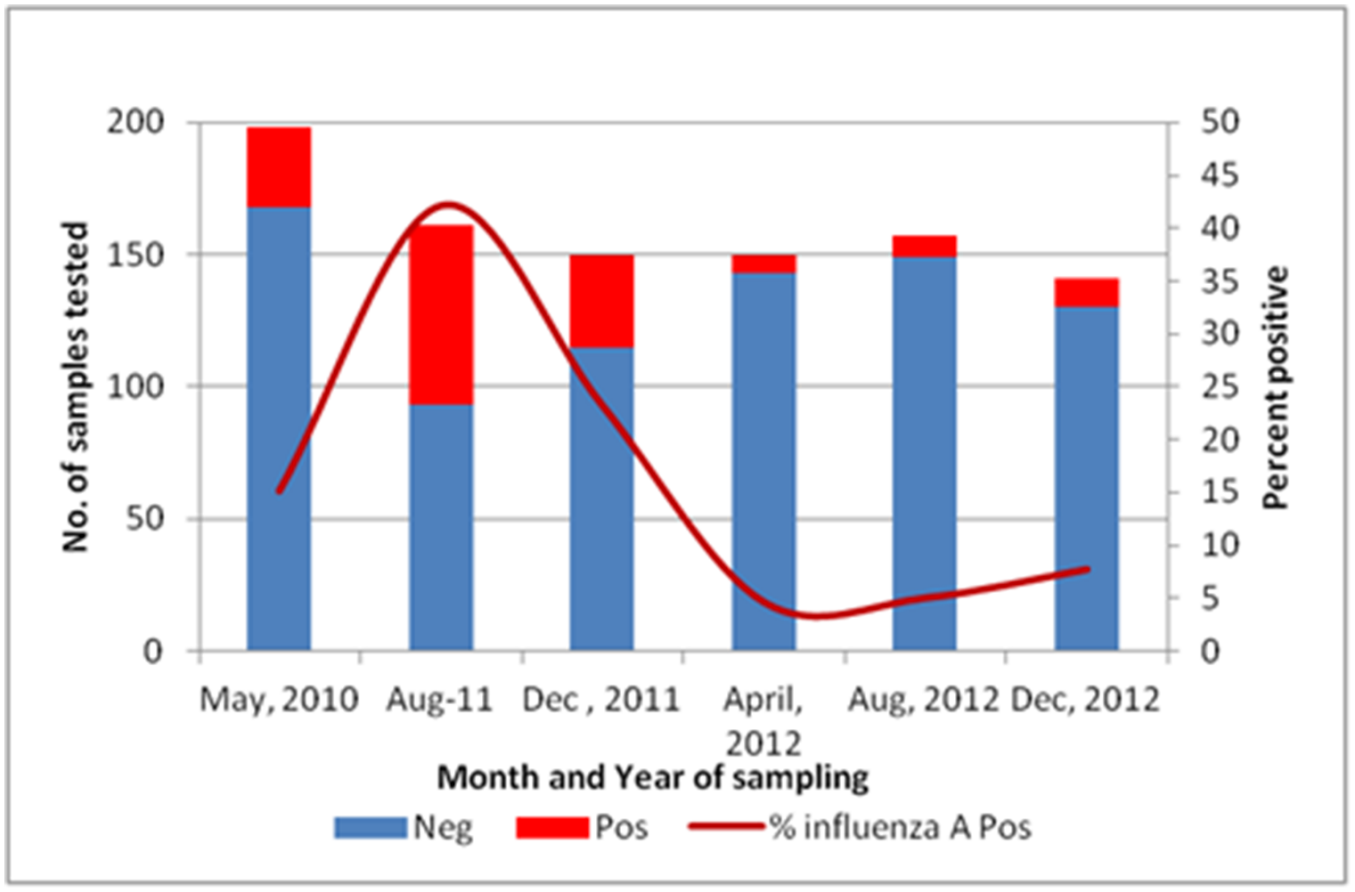
Influenza A seroprevalence by ELISA among pigs sampled at the slaughter house, 2010–2012.

A total of 978 pigs were sampled. The median number of pigs delivered to the slaughterhouse from individual farms was 1 (range = 1–33). Of the 512 (52.3%) pigs sampled whose source district was known, 501(97.8%) were from Kiambu County. Of the 957 pigs from the slaughterhouse tested by ELISA for influenza A antibodies, 16.6% (n = 159) were positive. The seroprevalence varied throughout the year (Fig 2); with the highest seroprevalence of 42.2% in August 2011. Comparison of the observed sero-prevalence among the 6 periods shown in Fig 2 revealed statistically significant heterogeneity (χ^2^ = 119.8, df = 5, *p*<0.0001).

### Influenza A HI testing among household and slaughterhouse samples

A total of 176/181 (97%) of ELISA-positive sera from pigs = 172, dogs = 2 and one each of chicken and ducks were tested by HI. There was insufficient volume of serum for HI assay for one positive serum each from cat, chicken and dog.

#### Influenza A HI testing in pigs

The overall influenza A seroprevalence among pigs from the household and slaughterhouse was (15.9%; 172/1084). All ELISA-positive sera from pigs were tested by HI. Positive titers (HI titers ≥ 1:80) were obtained when sera was tested with A/California/04/2009(H1N1) [71.5% (n = 123)] A/Swine/Texas/4199-2/98 triple-reassortant H3N2 [20.9% (n = 36)] and A/Swine/Iowa/15/30 H1N1 [14.4% (n = 25)] antigens (Table 3). Nearly half of the sera (48.4%, n = 84) had positive titers to only one antigen (A/California/04/2009 (H1N1)), one sera had positive titers to only the A/Swine/Texas/4199-2/98 triple-reassortant H3N2, 23.8% (n = 41) of the sera had positive titers to two or all three antigens on the HI panel and 26.7% (n = 26) were negative to all three of the reference antigens. The proportion of ELISA-positive sera in pigs from which positive titers to A/California/04/2009(H1N1) were obtained was variable by year of sampling over this study period and declined from 97.1% in 2010 (34/35) to 68.5% (76/111) in 2011, and 52.0% (13/25) in 2012.

**Table 3:** Distribution of hemagglutination-inhibition (HI) titers and proportion positive of enzyme-linked immunosorbent assay (ELISA)-positive pig sera against three reference antigens for household and slaughterhouse sites, May 2010 –August 2012 (n = 172)*.

Reference Viruses	Number with HI antibody titers (%)						Number n(%) of positive^†^ samples
Titers	≤40‡	80	160	320	640	1280	
A/California/04/2009(H1N1)	49(28.5)	24(13.9)	35(20.3)	35(20.3)	21(12.2)	8(4.6)	123(71.5)
A/Swine/Texas/4199-2/98 TR H3N2	13679.1	31(18.0)	3(1.7)	2(1.5)	0(0.0)	0(0.0)	36(20.9)
A/Swine/Iowa/15/30H1N1	1485.5	18(10.5)	5(2.9)	1(0.6)	1(0.6)	0(0.0)	25(14.5)

*included 13/16 positive from households and 159 from slaughterhouse

^†^ HI titers ≥ 1:80 were considered positive

^‡^ combined sera with HI titers 1:10 and 1:<10

#### Influenza A HI testing in dogs

Two of the three ELISA-positive dog sera were tested by HI, one was positive (HI titer 160) for A/California/04/2009(H1N1) and one was negative (HI titer 1:<10).

#### Influenza A HI testing in poultry

The duck and chicken sera were negative for A/California/04/2009(H1N1), HI titers 1:10 and 1:40 respectively.

### Influenza A virus detection

A total of 6289 [2,634 (41.9%) swabs from Kibera, 2,309 (36.7%) swabs from Asembo, and 1346 (21.4%) swabs from the slaughter house (collected from pigs) were screened for influenza A by rRT-PCR. The highest proportion of positive swabs was reported in ducks at 1.2% (5/423) followed by dogs 1%(4/400), pigs 0.7% (11/1491), and 0.6% in chickens (24/3863). None of the samples collected from turkeys (n = 33) and cats (n = 79) were positive (Table 4).

**Table 4:** Prevalence of PCR positivity for influenza A in nasal, oropharyngeal and cloacal swabs, by species and site, Kenya, May 2010-August 2012.

Species	Study site						Total		
	Kibera		Asembo‡		Slaughter house				
	Total samples tested	No. positive n (%)	Total samples tested	No. positive n (%)	Total samples tested	No. positive n (%)	Total samples tested	No. positive n (%)	Binomial Exact 95% CI
Cat	53	0(0)	26	0(0)	-	-	79	0(0)	5.4*
Chicken	1865	23(1.2)	1998	1(0.1)	-	-	3863	24(0.6)	0.3, 0.9
Dog	181	1(0.6)	219	3(1.4)	-	-	400	4(1.0)	0.3, 2.5
Duck	371	5(1.3)	52	0(0)	-	-	423	5(1.2)	0.4, 2.7
Pig	145	4(2.7)	0	0(0)	1346	7(0.5)	1491	11(0.7)	0.4, 1.3
Turkey	19	0(0)	14	0(0)	-	-	33	0(0)	12.4*
**Total**	**2634**	**33(1.3)**	**2309**	**4(0.2)**	**-**	**-**	**6289**	**44(0.7)**	**0.5, 0.9**

^‡^ In Asembo samples were not collected in May 2010

*one-sided, 97.5% confidence interval reported

### Influenza virus isolation

Virus isolation in MDCK cells was attempted for 13 of 15 influenza A positive swabs from pig (n = 11) and dog (n = 2) and 39 influenza A negative pig nasal swabs. Eight (21.6%) virus isolates were obtained from influenza A positive pig nasal swabs collected in July 2011 in Kibera (n = 5) and among slaughterhouse pigs collected in August 2011 (n = 3). All eight isolates were identified as A (H1N1)pdm09 on subtyping using rRT-PCR, and full genome sequencing was conducted on four randomly selected isolates (Table 5). No virus growth was observed from the two dog nasal swabs and the rRT-PCR-negative swabs from pigs.

**Table 5:** List of the Kenya swine isolates strain names and GenBank accession numbers.

Date of collection	Site collection	Strain Name	GenBank accession number for segments (S1-S8, respectively) submitted
7/25/2011	Kibera	A/swine/Kenya/9455/2011	KJ680515, KJ680519, KJ680523, KJ680527, KJ680531, KJ680535, KJ680539, KJ680543
7/25/2011	Kibera	A/swine/Kenya/9469/2011	KJ680516, KJ680520, KJ680524, KJ680528,KJ680532, KJ680536, KJ680540, KJ680544
7/25/2011	Kibera	A/swine/Kenya/9470/2011	KJ680517, KJ680521,KJ680525, KJ680529,KJ680533, KJ680537, KJ68054, KJ680545
8/5/2011	Ndumbu-ini slaughterhouse	A/swine/Kenya/1613/2011	KJ680514, KJ680518, KJ680522, KJ680526, KJ680530, KJ680534, KJ680538, KJ680542

Virus isolation in embryonated chicken eggs was attempted in all 29 Influenza A positive chicken and duck swabs [chicken n = 24 an duck n = 5] and 10 negative chicken swabs. Virus growth (positive for hemagglutination) was observed in one (3.4%) positive swab on the second passage but was negative for influenza A on post culture rtRTPCR.

### Phylogenetic analysis of the hemagglutinin (HA) gene

**Fig 3:**
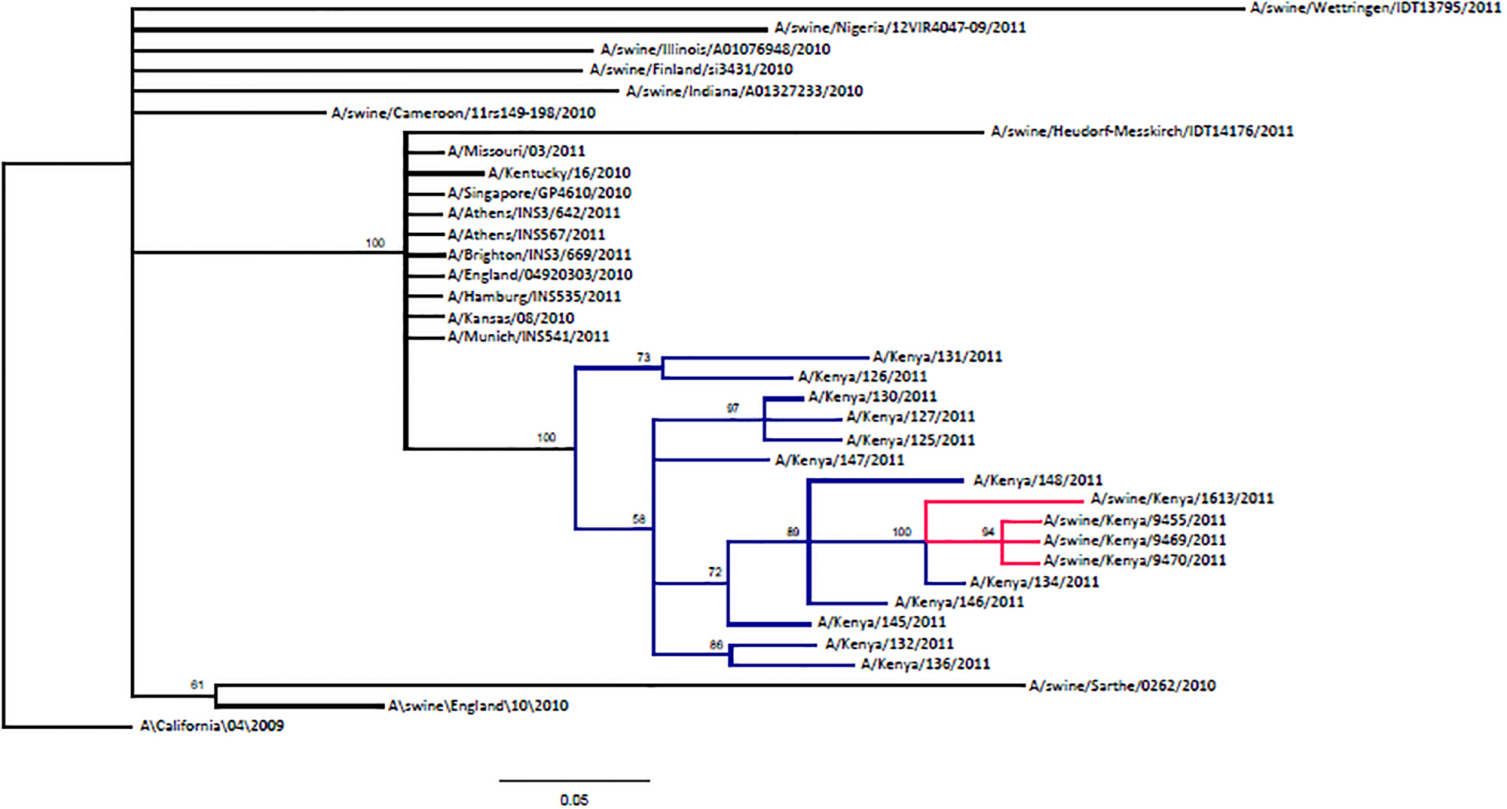
Analysis of HA segment of Kenya swine isolates alongside other contemporary isolates. Branches highlighted in red referto Kenya swine isolates while those in blue are Kenya human isolates. A/California/04/2009 is used to root the phylogenetic tree.

For the HA genes, 24 human-origin sequences with high identity (approximately 99%) were downloaded from GenBank and aligned alongside the swine sequences. Of these, 12 were from Kenya, while seven from Europe, four from North America and one from Thailand. In addition, nine swine isolates from Africa (n = 2), North America (n = 2) and Europe (n = 5) were included in the phylogenetic analysis (S2 Table). The HA segment of Kenyan swine isolates showed the closest nucleotide sequence homology with Kenyan human AH1N1 pandemic viruses isolated in 2011 (Fig 3). These swine isolates as well as Kenya human isolates branched from human isolates from Europe, North America and Asia. However, the swine virus isolates were more distant from the two African swine isolates from Nigeria and Cameroon.

## Discussion

We report the isolation of A(H1N1)pdm09-like influenza virus from pigs following its introduction in humans in Kenya in July 2009 at the height of the pandemic [18]. Molecular analysis of the hemagglutinin genes suggested that the virus was introduced into the pig population from the human population in Kenya, a finding that has been frequently observed in other countries [19]. There was a high nucleotide sequence homology between HA genes of the swine and the human A (H1N1) pdm09 viruses circulating in 2011 in Kenya, compared to influenza viruses isolated elsewhere. This observation is similar to reports from Brazil, Canada, USA and China, [19, 20] suggesting local transmission of pandemic influenza virus from humans to animals in different ecosystems.

In this study, pigs showed a higher influenza A seroprevalence (16.1%) than was found in dogs, cats, chickens and ducks (all <2%). The seroprevalence observed in chickens (0.1%), ducks (1.2%) and turkeys (0%) in this study was comparable to the seroprevalences reported for the same period in Uganda among the same poultry types [21]. However this study reported a higher seroprevalence among pigs compared to 4.6% reported in Uganda [21]. Influenza A prevalence from oropharyngeal/cloacal swabs in chickens (0.6%) was comparable to that reported among chickens in Uganda (0.4%), and in a previous study in Kenya (0.8%) [5]. However, our data showed a lower prevalence in pigs, ducks and turkeys compared to the study in Uganda [21].

Over half (71.5%) of seropositive pigs showed evidence of previous exposure to A/California/04/2009(H1N1) suggesting that after the initial introduction, the pandemic virus strain could have become established in pig populations. In addition, there was persistent seroprevalence of A/California/04/2009(H1N1) in five of six sampling periods, similar to observations in other countries where the initial introduction was often accompanied by establishment of the new strain in swine populations and co-circulation with swine influenza strains [19, 22–24]. Serological evidence suggesting previous infection with the North American swine H3N2 and the H1N1 used in this study was unexpected but is similar to a recent study in pigs in West and Central Africa [25]. The authors noted that these viruses have not been reported among pigs in Africa.

Our study had some limitations. Over a quarter (26.7%) of the pig seropositive sera was not reactive to any of the antigens used in the HI assays. Lack of data on swine influenza viruses circulating in pigs in Kenya and other African countries limited selection of appropriate antigens for complete serology [25, 26]. Our findings of serologic reactivity to the swine H3N2 and H1N1 viruses may be due to cross-reactivity with other human or swine viruses. Serological cross-reactivity between some European and North American swine A(H1N1) and A(H1N1)pdm09 has been demonstrated [22]. However, we used a high cut-off titer (≥1:80) in comparison to other studies [22] to reduce the chance of detecting cross-reactive antibodies. We did not collect clinical data from the animals in the study and cannot therefore examine the association of clinical signs with influenza infection. Another limitation is that we did not collect data on age of the pigs sampled hence association of the serology results and age could not be made. However, most of the pigs presented for slaughter in this slaughterhouse are mature aged from 16 weeks to 2 years depending on the production system [27]. However, since pigs in Kenya are not vaccinated against swine influenza, the antibodies detected were from natural infections.

Pigs are known to play an important role in the epidemiology of influenza A viruses in general and the A(H1N1)pdm09 virus specifically [2, 28]. Our findings highlight the need for continued monitoring of influenza strains circulating in pigs as well as the study of transmission of influenza strains between humans and pigs in this region. These findings contribute to our understanding of the epidemiology of influenza viruses in pigs in Africa, where little or no surveillance is currently being carried out on this disease.

## Supporting information

S1 Table Primer sets for PCR amplification and sequencing of the full genome of Kenya swine influenza A isolates. (DOCX)

S2 Table List of influenza virus isolates included in the phylogenetic analysis of the hemagglutinin segment. (DOCX)
