# Supplementary material for: Identification and characterization of influenza A viruses in selected domestic animals in Kenya, 2010-2012
Source: PLoS One. 2018 Feb 9;13(2):e0192721. doi: 10.1371/journal.pone.0192721 (PMC5806879; doi:10.1371/journal.pone.0192721)
Supplement: S1 Table — (DOCX) [file pone.0192721.s001.docx]

S1Table: Primer sets for PCR amplification and sequencing of the full genome of Kenya swine influenza A isolates.

| **Gene** | **Segment** | **Forward sequence (5' to 3')** | **Reverse sequence (5' to 3')** |
| --- | --- | --- | --- |
| PB2 | 1 | tgtaaaacgacggccagtttgaatggatgtcaatccga | caggaaacagctatgacccttccatrattacatcytgtg |
|  | 2 | tgtaaaacgacggccagtgtracatggtggaayagaa | caggaaacagctatgaccgctttgrtcaayatcrtcatt |
|  | 3 | tgtaaaacgacggccagtcctggtcaygcagacctcag | caggaaacagctatgaccccaaarctgaaggaygarctgat |
|  | 4 | tgtaaaacgacggccagtcaagcagtrtrtacattgaagt | caggaaacagctatgacccctctaactgcttttaycatgcaat |
|  | 5 | tgtaaaacgacggccagtccracwgaagaacaagctgt | caggaaacagctatgaccggagtattcatcyacacccat |
|  | 6 | tgtaaaacgacggccagtaagcaaccagragattgrttca | caggaaacagctatgaccctgagaccaytgaattttraca |
|  | 7 | tgtaaaacgacggccagtccaagyacmgagatgtcaatgaga | caggaaacagctatgaccttrctcarttcattgatgct |
|  | 8 | tgtaaaacgacggccagtcaatacctaycartggatcatcagaa | caggaaacagctatgacctagtagaaacaaggtcgtt |
| PB1 | 1 | tgtaaaacgacggccagtagcaaaagcaggtcaatt | caggaaacagctatgaccctraawacttctatrgtgtt |
|  | 2 | tgtaaaacgacggccagtcaactcaacccrattgatggrccact | caggaaacagctatgaccgttcaagcttttcrcawatg |
|  | 3 | tgtaaaacgacggccagtacaagrgtggacaaatrac | caggaaacagctatgaccctgaaccaytcaggytgattt |
|  | 4 | tgtaaaacgacggccagttgaacacratgaccaarga | caggaaacagctatgaccttgaacatgcccatcatcatyccagg |
|  | 5 | tgtaaaacgacggccagtaatcaaaaycctmgaatgtt | caggaaacagctatgaccagctccatgctraaattrgc |
|  | 6 | tgtaaaacgacggccagtcaaataccygcagaratgctagc | caggaaacagctatgaccccaagrtcattgtttatcat |
|  | 7 | tgtaaaacgacggccagtatgagyaaaaagaagtcyta | caggaaacagctatgacctcaatytcyttatggctgac |
|  | 8 | tgtaaaacgacggccagtgcyaatttyagcatggagct | caggaaacagctatgaccagtagaaacaaggca ttt |
| PA | 1 | tgtaaaacgacggccagtagcaaaagcaggtactgat | caggaaacagctatgacctartckgcctttgtggccatttc |
|  | 2 | tgtaaaacgacggccagtccaaatgcactkttaaarcacagatt | caggaaacagctatgacctgagaaagcttgcctcaatg |
|  | 3 | tgtaaaacgacggccagttatgaytacaargagaa | caggaaacagctatgaccggttctttccatccaaagaatgtt |
|  | 4 | tgtaaaacgacggccagttgcmttgaraattttagraccta | caggaaacagctatgacctcrcakgccttgttgaactcatt |
|  | 5 | tgtaaaacgacggccagtaaattragcattgargaycca | caggaaacagctatgacctcwagtctygggtcagtgag |
|  | 6 | tgtaaaacgacggccagttaagcgatttraagcaatatga | caggaaacagctatgaccaayccytcyaattgtggwgatg |
|  | 7 | tgtaaaacgacggccagtaatgcatcctgtgcagcaatgga | caggaaacagctatgaccttgtccctaagagcctgaacaa |
|  | 8 | tgtaaaacgacggccagtatgaartggggaatggagatgag | caggaaacagctatgaccagtagaaacaaggtacctttt |
| HA | 1 | tgtaaaacgacggccagtatacgactagcaaaagcagggg | caggaaacagctatgacctcatgattgggccayga |
|  | 2 | tgtaaaacgacggccagtacrtgttacccwggrgatttca | caggaaacagctatgaccgaaakgggagrctggtgttta |
|  | 3 | tgtaaaacgacggccagtatgargarctragagagca | caggaaacagctatgacccaatggcrttytgtgtgctc |
|  | 4 | tgtaaaacgacggccagtagratgractattactggac | caggaaacagctatgaccttctkcattrtawgtccaaa |
|  | 5 | tgtaaaacgacggccagttggatggtayggttaycayca | caggaaacagctatgacctcataagtyccatttytga |
|  | 6 | tgtaaaacgacggccagtaagatgaayacrcarttcacag | caggaaacagctatgaccgtgtcagtagaaacaagggtgttt |
| NP | 1 | tgtaaaacgacggccagtcagggtagataatcactcac | caggaaacagctatgaccagagcacatyctgggatccat |
|  | 2 | tgtaaaacgacggccagtatggtrctctctgcttttgatga | caggaaacagctatgacctttgtgcagctgtttgaaatttyccttt |
|  | 3 | tgtaaaacgacggccagttggcattchaatttraatgat | caggaaacagctatgaccctgrctcttgtgtgcdgg |
|  | 4 | tgtaaaacgacggccagtgctgcagtcaarggart | caggaaacagctatgaccaagcratttgtacycctctagt |
|  | 5 | tgtaaaacgacggccagtcctgcytgtgygtawggac | caggaaacagctatgaccagtagaaacaagggtatttttc |
| NA | 1 | tgtaaaacgacggccagtagcaaaagcaggagt | caggaaacagctatgaccctggaccrgaaattcc |
|  | 2 | tgtaaaacgacggccagttacacaaaagacaayagc | caggaaacagctatgaccggrccatcggtcattatg |
|  | 3 | tgtaaaacgacggccagtggtcagcaagcgcatgycatga | caggaaacagctatgacccatatytgtatgaaaacc |
|  | 4 | tgtaaaacgacggccagtaatggrcargcctcrtacaa | caggaaacagctatgaccgctgctyccrctagtccagat |
|  | 5 | tgtaaaacgacggccagttaggatacatctgcagtgg | caggaaacagctatgaccagtagaaacaaggag |
| M | 1 | tgtaaaacgacggccagtagcaaaagcaggtag | caggaaacagctatgaccgcaatctgytcacakgt |
|  | 2 | tgtaaaacgacggccagtcaccgtgcccagtgagcg | caggaaacagctatgacctcayttgaaycgytgcat |
|  | 3 | tgtaaaacgacggccagttctgctggwgcacttgccagttg | caggaaacagctatgaccagtagmaacaaggtagt |
| NS | 1 | tgtaaaacgacggccagtagcaaaagcagggtgacaaagaca | caggaaacagctatgacctcggtgaaagccctta |
|  | 2 | tgtaaaacgacggccagttgaggcwyttaaaatgacca | caggaaacagctatgaccagtagaaacaagggtgttttttat |
|  | 3 | tgtaaaacgacggccagtaaagcdaayttcagtgtg | caggaaacagctatgaccttcaathagccatctta |
