# Supplementary material for: Identification and characterization of influenza A viruses in selected domestic animals in Kenya, 2010-2012
Source: PLoS One. 2018 Feb 9;13(2):e0192721. doi: 10.1371/journal.pone.0192721 (PMC5806879; doi:10.1371/journal.pone.0192721)
Supplement: S2 Table — (DOCX) [file pone.0192721.s002.docx]

S2 Table: List of influenza virus isolates included in the phylogenetic analysis of the hemagglutinin segment

| No | Strain Name | Subtype | Segment | Country | Host species | Year of  Collection | GenBank accession number (Reference) |
| --- | --- | --- | --- | --- | --- | --- | --- |
| 1 | A/Kenya/125 | H1N1 | HA | Kenya | Human | 2011 | JQ396224 |
| 2 | A/Kenya/126 | H1N1 | HA | Kenya | Human | 2011 | JQ396225 |
| 3 | A/Kenya/127 | H1N1 | HA | Kenya | Human | 2011 | JQ396226 |
| 4 | A/Kenya/130 | H1N1 | HA | Kenya | Human | 2011 | JQ396229 |
| 5 | A/Kenya/131 | H1N1 | HA | Kenya | Human | 2011 | JQ396230 |
| 6 | A/Kenya/132 | H1N1 | HA | Kenya | Human | 2011 | JQ396231 |
| 7 | A/Kenya/134 | H1N1 | HA | Kenya | Human | 2011 | JQ396232 |
| 8 | A/Kenya/136 | H1N1 | HA | Kenya | Human | 2011 | JQ396233 |
| 9 | A/Kenya/145 | H1N1 | HA | Kenya | Human | 2011 | JQ396238 |
| 10 | A/Kenya/146 | H1N1 | HA | Kenya | Human | 2011 | JQ396239 |
| 11 | A/Kenya/147 | H1N1 | HA | Kenya | Human | 2011 | JQ396240 |
| 12 | A/Kenya/148 | H1N1 | HA | Kenya | Human | 2011 | JQ396241 |
| 13 | A/swine/Cameroon/11rs149-198 | H1N1 | HA | Cameroon | Swine | 2010 | JF707784 |
| 14 | A/swine/England/10 | H1N1 | HA | United Kingdom | Swine | 2009 | CY115943 |
| 15 | A/swine/Finland/si3431 | H1N1 | HA | Finland | Swine | 2009 | KC336410 |
| 16 | A/swine/Heudorf-Messkirch/IDT14176/2011 | H1N1 | HA | Germany | Swine | 2010 | KC631888 |
| 17 | A/swine/Illinois/A01076948 | H1N1 | HA | USA | Swine | 2010 | CY114613 |
| 18 | A/swine/Indiana/A01327233 | H1N1 | HA | USA | Swine | 2010 | JX463281 |
| 19 | A/swine/Nigeria/12VIR4047-09 | H1N1 | HA | Nigeria | Swine | 2011 | JX442481 |
| 20 | A/swine/Sarthe/0262 | H1N1 | HA | France | Swine | 2011 | FR871195 |
| 21 | A/swine/Wettringen/IDT13795 | H1N2 | HA | Germany | Swine | 2010 | KC222548 |
| 22 | A/Athens/INS3_642 | H1N1 | HA | Greece | Human | 2011 | CY176482 |
| 23 | A/Athens/INS567 | H1N1 | HA | Greece | Human | 2011 | CY129467 |
| 24 | A/Brighton/INS3_669 | H1N1 | HA | United Kingdom | Human | 2011 | CY176405 |
| 25 | A/California/04 | H1N1 | HA | USA | Human | 2009 | FJ966082 |
| 26 | A/England/04920303 | H1N1 | HA | United Kingdom | Human | 2010 | JX625758 |
| 27 | A/Hamburg/INS535 | H1N1 | HA | Germany | Human | 2011 | CY129598 |
| 28 | A/Kansas/08 | H1N1 | HA | USA | Human | 2010 | KC882013 |
| 29 | A/Kentucky/16 | H1N1 | HA | USA | Human | 2010 | KC882263 |
| 30 | A/Missouri/03 | H1N1 | HA | USA | Human | 2011 | KC882295 |
| 31 | A/Munich/INS541 | H1N1 | HA | Germany | Human | 2011 | CY129638 |
| 32 | A/Singapore/GP4610 | H1N1 | HA | Singapore | Human | 2011 | CY091674 |
